# Non-contact method to freely control the radiation patterns of antenna with multi-folded transformation optics

**DOI:** 10.1038/s41598-017-13318-y

**Published:** 2017-10-13

**Authors:** Hamza Ahmad Madni, Bin Zheng, Rongrong Zhu, Lian Shen, Hongsheng Chen, Zhiwei Xu, Shahram Dehdashti, Yaodong Zhao, Huaping Wang

**Affiliations:** 1 The Innovative Institute of Electromagnetic Information and Electronic Integration, Department of Electronic Engineering, Zhejiang University, Hangzhou, 310027 China; 2 Science and Technology on Electronic Information Control Laboratory, Chengdu, 610036 China; 3 Institute of Marine Electronics Engineering, Zhejiang University, Hangzhou, 310058 China; 4 Department of Computer Engineering, Khwaja Fareed University of Engineering & Information Technology, Rahim Yar Khan, 64200 Pakistan

## Abstract

In this paper, we propose to use multi-folded transformation optics method to design a non-contact illusion device that can distantly and freely manipulate the radiation behavior of antenna located at a certain distance and such manipulation is enabled by the use of mapped electromagnetic medium coated with the transformed medium. The proposed design aims to achieve the radiation pattern of our choice from the antenna that does not possess any electromagnetic medium. Based on this, the functionality of parabolic antenna is distantly achieved from the point source. We further extended our idea to array of antennas in which the proposed device distantly makes the linear array of antennas behave like a geometrically different array of antennas. Our work extends the concept of illusion optics for active scatterer that will be very helpful for future antenna design.

## Introduction

Transformation optics (TO) has now attracted many peoples’ interest due to the manipulation feature of the electromagnetic (EM) waves in a user-defined manner. Ever since the proposal reported in^1,2^, the TO concept has motivated a series of studies on other functional and conceptual devices^3–22^ in the field of wave-guiding^3–8^ and in the field of antennas and lenses such as: focusing devices^9–11^, directive antennas^12–14^, multi-beam^15–17^, and isotropic emissions^18,19^. Moreover, the techniques of source transformation^23–28^ have opened new ways for the design of active devices with source distribution recessed in the transformed space.

Apart from other TO-based devices, the illusion devices^29–37^ are also important that can fool the viewer (or detector) into making the wrong decisions. For example, an object can be made to appear like another one^29,30^. Similarly, the illusion devices for active scatterer are capable of producing an illusion on antenna’s radiation pattern^36,37^. Up to now, the key feature of all the previously proposed illusion devices for active scatterer is that the emission point is either fully recessed or bounded by the properly designed EM medium. In this scenario, the coated EM medium is good to protect the active sources but it might prevent the matter exchange between the antennas and the external environment. Realistically, it remains a big challenge to achieve the desired wave fronts when the radiating elements are not recessed or bounded with any EM medium.

Now the question raised that whether we can modify the EM sources and virtually delocalize the emission point when the active sources are unbounded with EM medium. In other words, is it possible to distantly generate the EM radiation of the “naked” radiating elements such that we have the impression that the emission like coming from a virtual source at another location or where no radiator is physically present?

In this paper, we use transformation EM to prove that it is possible to generate the desired emission from the “naked” source where no actual source is physically present. In this regard, we introduced the scheme of non-contact illusion device with the ability of achieving the desired radiation patterns from antenna while the antenna is unbounded with any EM medium. The proposed non-contact device performs as a bridge to efficiently link the radiating waves with the antenna, which has potential to fool radar detectors. Whereas, the required constitutive parameters of the proposed device can thus be obtained by using multi-folded TO method^30,38,39^. In this way, the guidance of radiation pattern through a certain distance can be achieved by embedding a mapped EM medium into a transformed medium^40^ whose constitutive parameters are obtained by judiciously squeezing the larger space excluding antenna aperture into a compressed region. The beauty of the proposed design is that the transformed medium at a certain distance will behave exactly the same as the original EM medium bounded with the antenna.

To understand how the radiation pattern of source can be distantly and freely manipulated, we will consider three different examples. In the first example, a simple antenna source is manipulated to achieve the desired radiation pattern while the antenna source is not physically in contact with the proposed device. In this case, the observer will have the impression that the emission is coming from a different position instead of its real physical location. Based on this, the proposed non-contact device is used to achieve the functionality of parabolic antenna^25^ from the point source. Finally yet importantly, the proposed device enables the array of antennas to behave like a geometrically different array of antennas. The proposed non-contact concept is devoted to create illusions for active scatterer (antenna) and in each example; the designed device is placed at a certain distance from the active sources. The proposed concept can enhance the potential of antennas for remote controlling applications and in the other fields of engineering. Full wave finite element method is used to verify the expected behavior of our proposed concept in order to distantly and freely manipulate the radiation patterns.

## Results and Discussion

**Figure 1:**
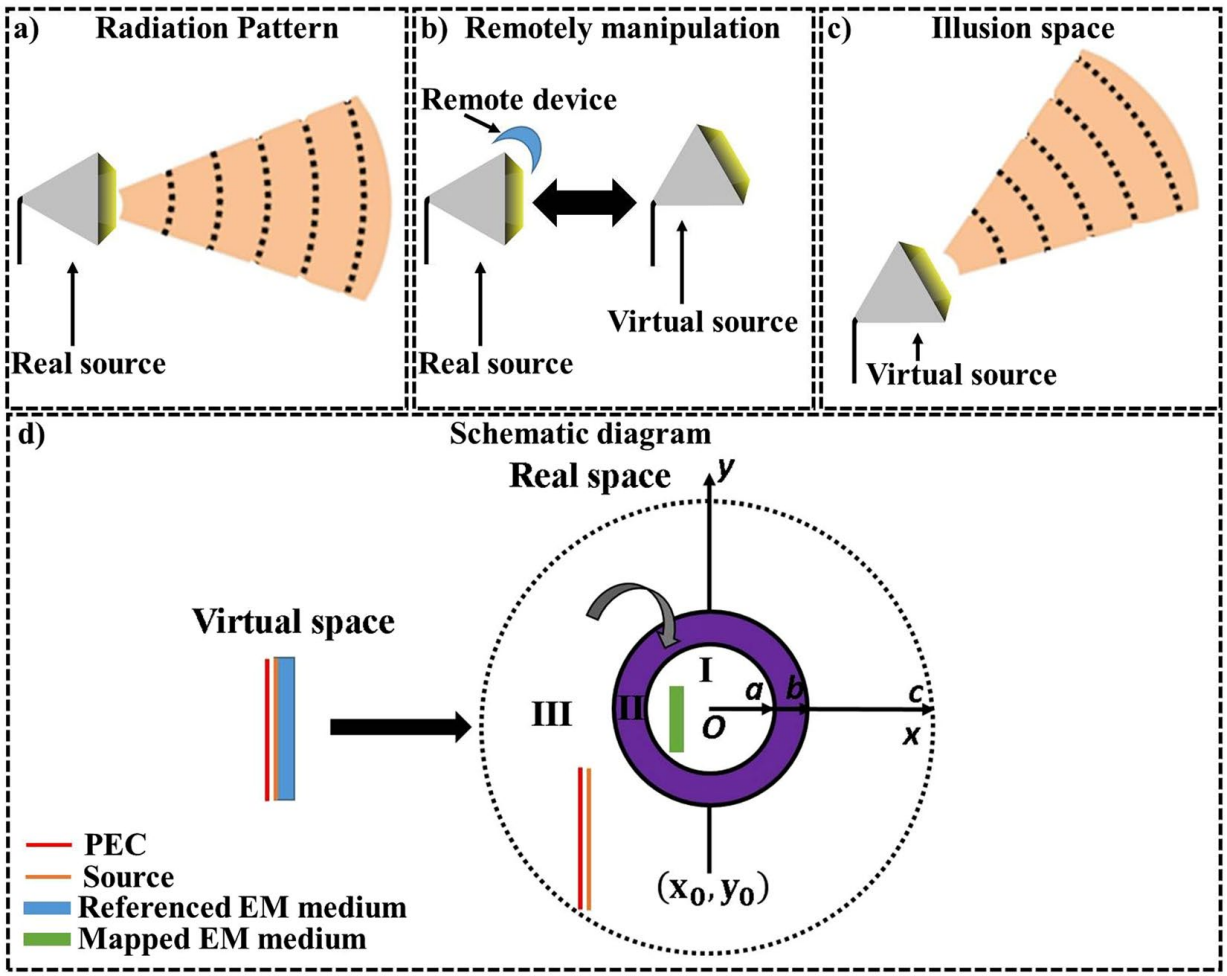
The working phenomenon of the non-contact illusion device that distantly delocalizes the emission of antenna into a desired manner. (**a**) The emission of the radiating antenna in free space is in forward direction, which is distantly manipulated by non-contact device (**b**). Observer outside the non-contact device has the impression that the radiation comes from another emitting antenna’s direction, which is optically equivalent to (**c**). (**d**) The schematic diagram of proposed non-contact device. In virtual space: current source, PEC and the referenced EM medium exist. After applying the transformation only referenced EM medium is compressed and shifted towards the compressed region I. Region I is further coated with the folding region II. In all cases, the linear source and PEC remains same.

Unlike previous reported works, in this paper we aim to create a link between the radiating elements and the proposed device at a distance, so that the emission can be distantly and freely manipulated, as summarized in Fig. 1. Therefore, the principle of the proposed device allows shifting the radiation pattern compare to the actual emission, as shown in Fig. 1(a). Indeed, the detector (or observer) looking at the radiation pattern of the source placed besides the non-contact device will observe a different radiation pattern. In this sense, the observer will have the impression that the emission comes from another source’s location, which can be seen in Fig. 1(b). In simple words, the proposed non-contact illusion device (Fig. 1(b)) makes the radiation pattern of Fig. 1(a) exactly the same as of Fig. 1(c).

The schematic diagram for the proposed non-contact device is shown in Fig. 1(d). In which, the yellow colored line source is physically attached with the blue colored EM medium (virtual space). Whereas, the red line besides the line source is perfect electric conductor (PEC) in order to achieve the radiation patterns only in forward direction. Next, in real space the black dashed line circle represents the virtual boundary, which is also covering the region of source. In that step, the circle of radius c (region III) is squeezed into region I, known as the compressed region. In this condition, the referenced EM medium in virtual space is also compressed and shifted to region I, which can be seen as a green colored segment. That compressed region (region I) is further coated with the complementary medium (region II) by folding the space $b < r < c$ into $a < r < b$, for more details please see method summary.

Here, we illustrate our method with the example that; initially we suppose that the referenced EM medium (blue colored) in virtual space is filled with air then after transformation the referenced EM medium will transform into a mapped EM medium (green colored). In this condition, the resultant device will behave like a concentrator^3^. Hereafter, the material change in the referenced EM medium will also influence the material change in the mapped EM medium. Theoretically, the dramatically changes of the material parameters of EM medium will change the radiation pattern of source^7^. Thus, in our case we assumed the constitutive parameters of referenced EM medium first as an inhomogeneous material and then change the mapped EM medium accordingly.

In the following, full wave simulations of finite element method (COMSOL) are performed by adopting the transverse electric (TE) mode at the frequency of $4GHz.$ The geometric structure parameter for virtual space, a large current of height $0.2\,m$ is placed at $(-0.175,0)$, whereas, the location of PEC is $(-0.176,0)$ and the height is similar as that of the current source. While the thickness of referenced EM medium substrate is $-0.025\,m$, height is $0.2\,m$ at the origin point $(-0.1625,0)$. Similarly, the geometric parameters for non-contact device: the radius of $c=0.2\,m$, $b=0.09\,m$, $a=0.08\,m$ with the origin point is $O(-0.07,0.06)$. The obtained material parameters for each region can be seen in method summary.

**Figure 2:**
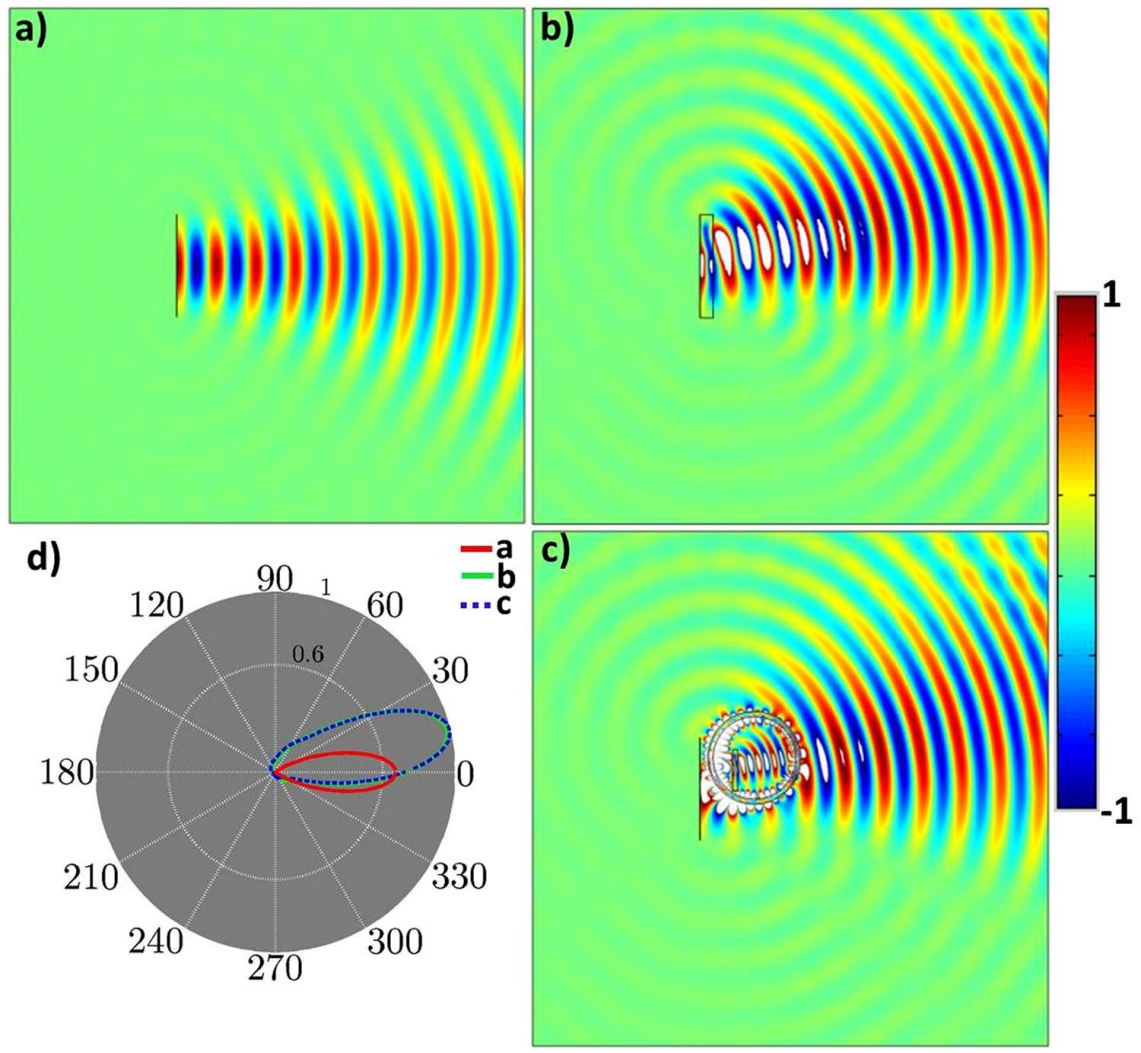
Simulation results. (**a**) z-directed electric field distribution of an electric field source with the magnitude of $\exp (-225{y}^{2})$ is placed in free space and its height is $0.2m$. The source is placed next to the PEC to achieve only forward-direction’s radiations. (**b**) z-directed electric field distribution of the similar source as of (**a**) with the referenced EM medium substrate of supposed materials i.e., ${\varepsilon }_{med}=40y+3$ and ${\mu }_{med}=1$. (**c**) z-directed electric field distribution of (**a**) is distantly changed as that of (b). (d) The corresponding radiation pattern of (**a**), (**b**), and (**c**).

We can now examine the functionality of both the original antenna with referenced EM medium and the proposed non-contact device. The radiated field emitted by the real source is presented in Fig. 2(a), and Fig. 2(b) shows the source of Fig. 2(a) is attached with the referenced EM medium that created different far-field radiation pattern. While Fig. 2(c) depicts the corresponding functionality of the proposed non-contact device with the similar current source as that of Fig. 2(a). As it is clearly illustrated that the real source is transformed into a virtual one, which is explained by the nature of transformation. As expected, for observers, the functionality of Fig. 2(c) and (b) are identical to each other, while the mapped EM medium in the non-contact device has been shrunken and far enough from the current source. Furthermore, Fig. 2(d) represents the corresponding normalized far-field radiation patterns of Fig. 2(a–c).

**Figure 3:**
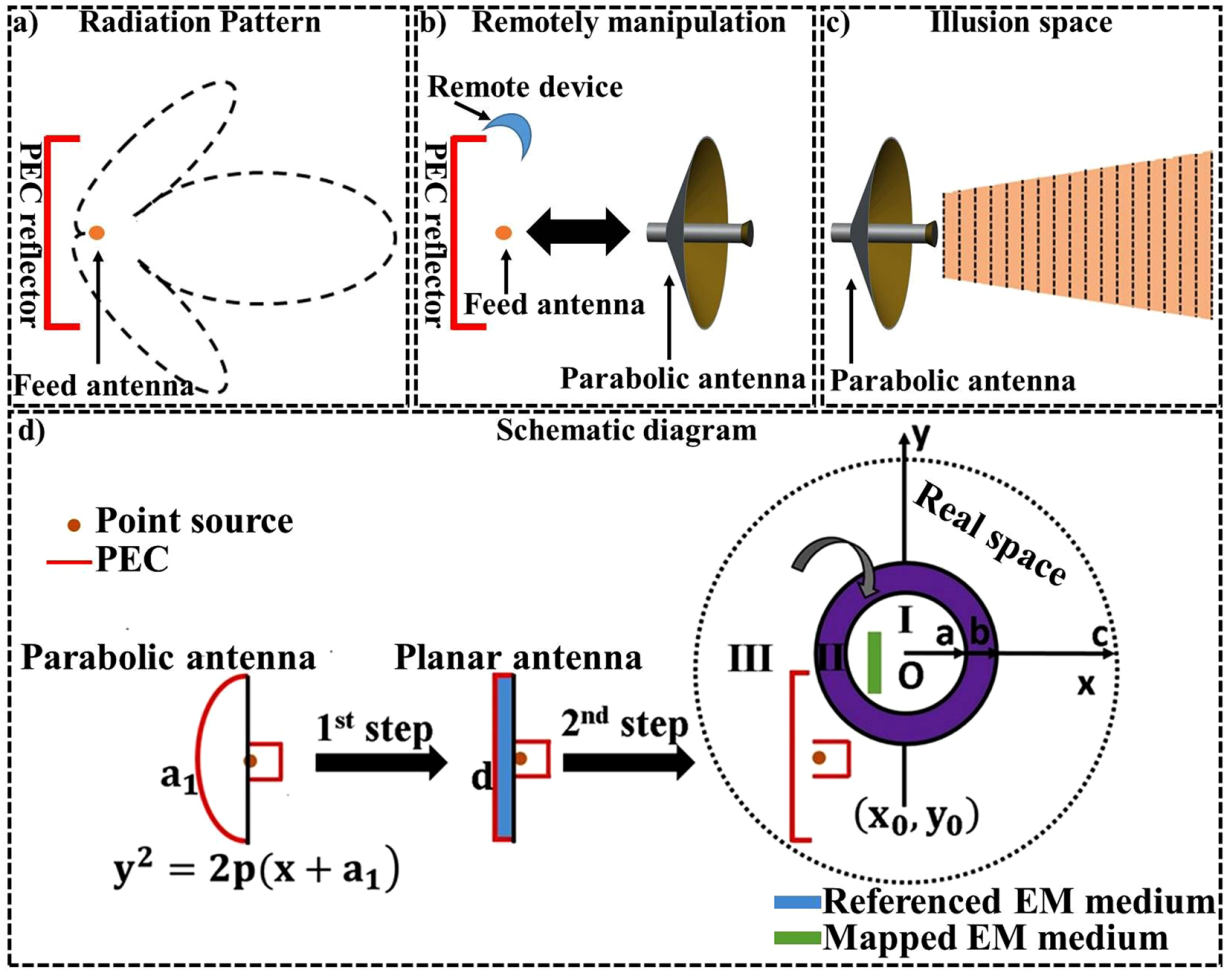
The working phenomenon of the non-contact illusion device that distantly modifies the source into a desired manner. (**a**) The emission of the feed antenna with PEC reflector in free space is in scattered form with larger side lobes, which is distantly manipulated by non-contact device (**b**). Observer outside the non-contact device has the impression that the radiation comes from another emitting antenna, which is optically equivalent to (**c**). (**d**) Schematic diagram of the proposed non-contact device. In first step, spatial coordinate transformation is applied to design a planar focusing antenna from virtual space to physical space^25^. The second step is achieved by recalling the Fig. 1(d). Red lines show PEC and yellow dot represents the feed antenna. In all cases, PEC reflector and the feed antenna remain same.

The simulation results of Fig. 2 have shown the possibility of distantly shifting the emission pattern (delocalization) without changing the source. Based on it, even more complex radiating manipulation can be performed. To further validate our approach, in Fig. 3(a) a PEC reflector with the feed antenna is shown. In this case, the wave fronts of the waves are scattered with higher side lobes (black-dashed lines). Anyway, again we ask question ourselves is whether we can distantly change the radiation pattern as if the emission is coming from a different source. For example, here, we are intended to transform the radiation pattern of the PEC reflector with feed antenna into a radiation pattern of parabolic antenna, as shown in Fig. 3(b). Whereas, Fig. 3(b) and (c) are optically equivalent.

In Fig. 3(d), we consider how to map the parabolic antenna into a planar antenna and then distantly change the radiation pattern of PEC reflector by recalling the Fig. 1(d). In the first step of Fig. 3(d), a parabolic antenna is transformed into the planar antenna^25^ with the parabolic equation ${y}^{2}=2p(x+{a}_{1})$. It should be noted that the red lines represent the PEC and yellow dot surrounded with PEC represent the horn feed antenna placed at the focus point of both in parabolic and planar antenna. The constitutive parameters for that particular step are obtained by ref.^25^. The resultant planar antenna looks similar as that of the virtual space in Fig. 1(d), so by taking advantage of this we further use second step to compress and shift the referenced EM medium into the compressed region I while, the PEC reflector and horn feed antenna will keep maintain their positions.

To verify the expected behavior of proposed non-contact planar device, simulations have been done in TE mode at the working frequency of $4GHz$. In the first step, the geometric structure of parabolic antenna with $p=0.1125$, ${a}_{1}=0.05625$ is compressed into $d=0.028125$ while $p$ will not change, whereas, the origin point is $(-0.1642,0)$. Similarly, in the second step, the geometric parameters for non-contact device: the radius of $c=0.21\,m$, $a=0.07\,m$, $b=0.075\,m$ with the origin point is $O(-0.05,0.05)$. The method to obtain material parameters is discussed in method summary.

**Figure 4:**
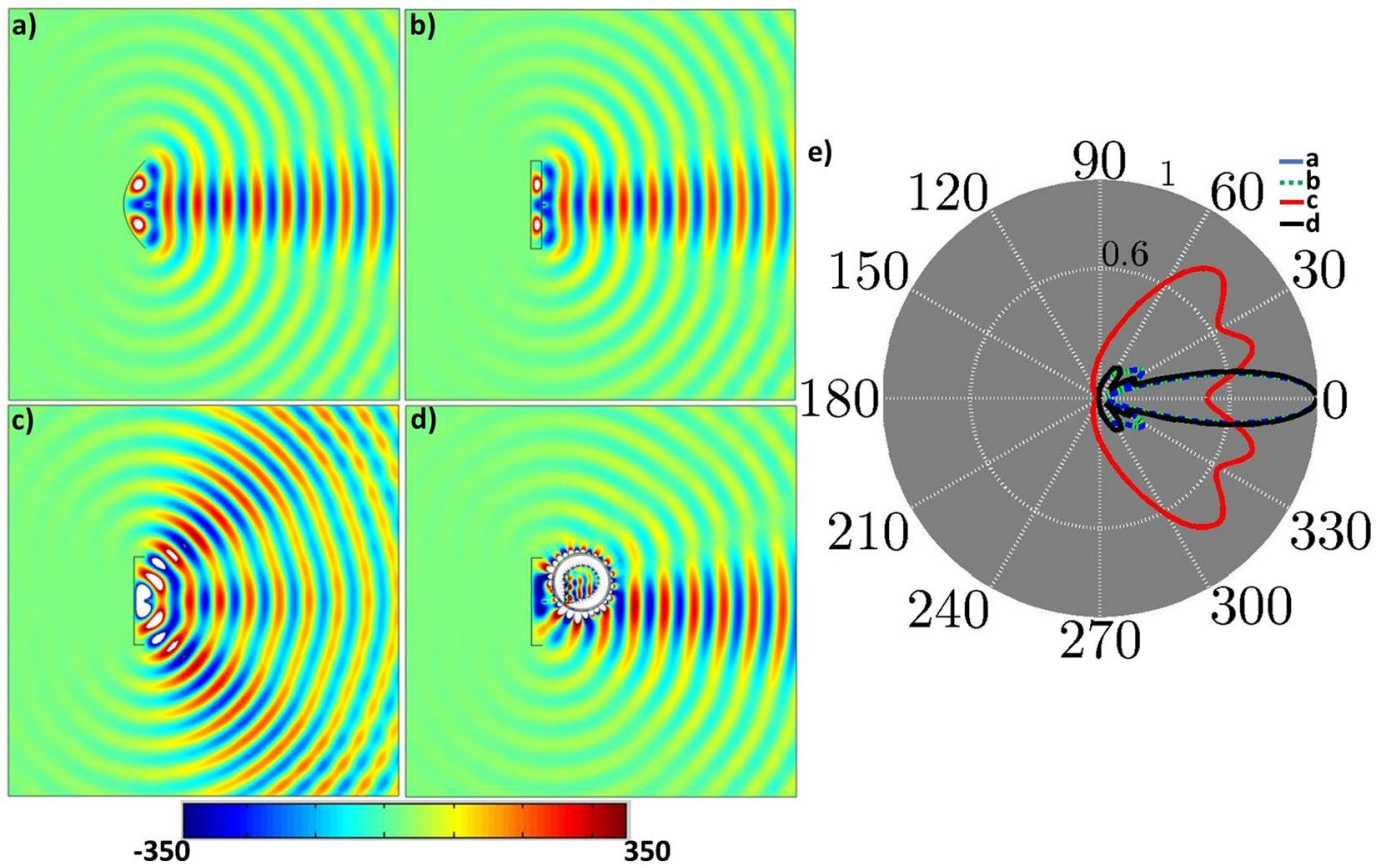
Simulation results. (**a**) z-directed electric field distribution of parabolic antenna and (**b**) is for planar antenna while (**c**) represents the PEC reflector with feed antenna in free space. (**d**) The non-contact device is used to distantly change the radiation pattern of (**c**) according to (**b**). (**e**) The normalized far-field radiation pattern of (**a**), (**b**). (**c**), and (**d**).

The simulation results are shown in Fig. 4(a–e). From the Fig. 4(a) (parabolic antenna) and 4(b) (planar antenna), one can see that the radiation patterns are identical to each other. Figure 4(c) represents the radiation patterns of only PEC reflector with the feed antenna. While in Fig. 4(d), the proposed non-contact planar device is used to minimize the side lobes of the source (Fig. 4(c)) and makes it similar as that of Fig. 4(b). Finally, the corresponding far-field radiation patterns are given in Fig. 4(e) for the Fig. 4(a,b,c and d).

**Figure 5:**
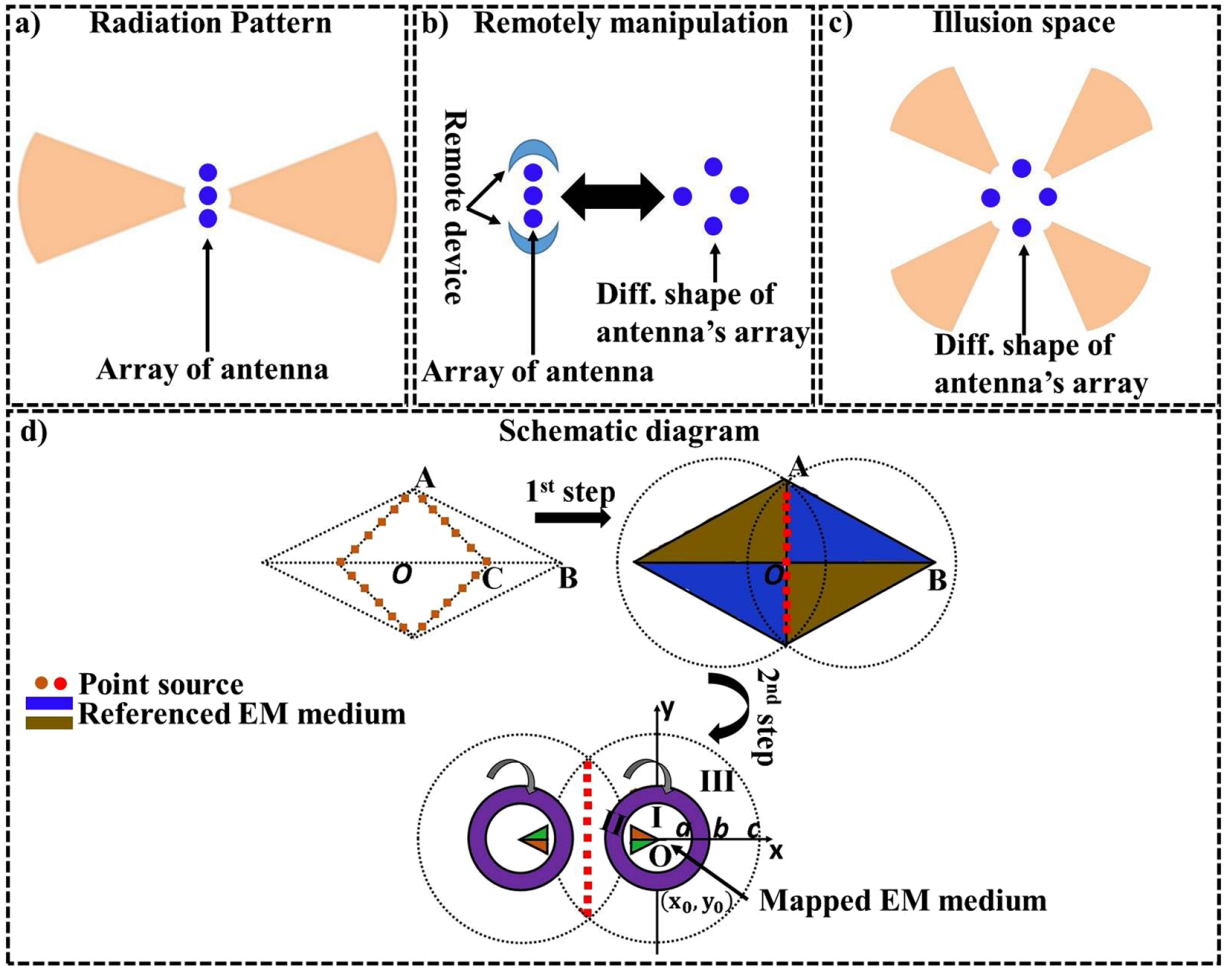
The working phenomenon of the non-contact illusion device that distantly modifies the array of sources into a desired manner. (**a**) The emission of the linear array of antennas is distantly manipulated in (**b**), so the observer outside the non-contact device has the impression that the radiation comes from different geometrical structure of antennas’ array, which is optically equivalent to (**c**). (**d**) Schematic diagram: in the first step, the brown colored array sources made of $N=22$ elements with the current density of each is $1/2$ are linearly transformed into a linear array made of $N=11$ elements with the current density of each is $1$, which can be seen in red colored. The second step is based on Fig. 1(d).

In the final example, suppose the linear array of antennas with their radiation pattern is shown in Fig. 5(a). It is also possible for antennas array of Fig. 5(a) to behave like a geometrically different array when surrounded by the proposed non-contact device, as shown in Fig. 5(b). For example, a linear antenna array surrounded by proposed device will behave like a diamond shape antenna array (Fig. 5(c)). Although, a simple case is treated here but according to the principle this scheme can be applied to arbitrarily complex arrays. The schematic diagram can be seen in Fig. 5(d) with two different steps and due to symmetric structure, here, the first quadrant portion is demonstrated for the first step. In the first step, the brown colored array sources made of $N=22$ elements are placed in virtual space are transformed into a linear array made of $N=11$ elements and can be seen in red colored. This transformation has been achieved by linearly expanding the region ${\rm{\Delta }}ABC$ to ${\rm{\Delta }}ABO$ (blue colored region). In the second step, the compression and folding technique is applied by recalling the Fig. 1(d) in order to achieve two elements of proposed non-contact device.

To verify the expected behavior of proposed non-contact array device, simulations have been done in TE mode at the working frequency of $4GHz$. The geometric structure for Fig. 5(d) is: $A(0,0.05)$, $B(0.1,0)$, $C(0.05,0)$, and for the second step: $O(0.1,0)$, $c=0.12$, $b=0.078$ and $a=0.0507$. Moreover, the current density of each element in virtual space (diamond shaped) is $1/2$ and after transformation it will becomes $1$ for linear array. The obtained material parameters of each step are mentioned in method summary.

**Figure 6:**
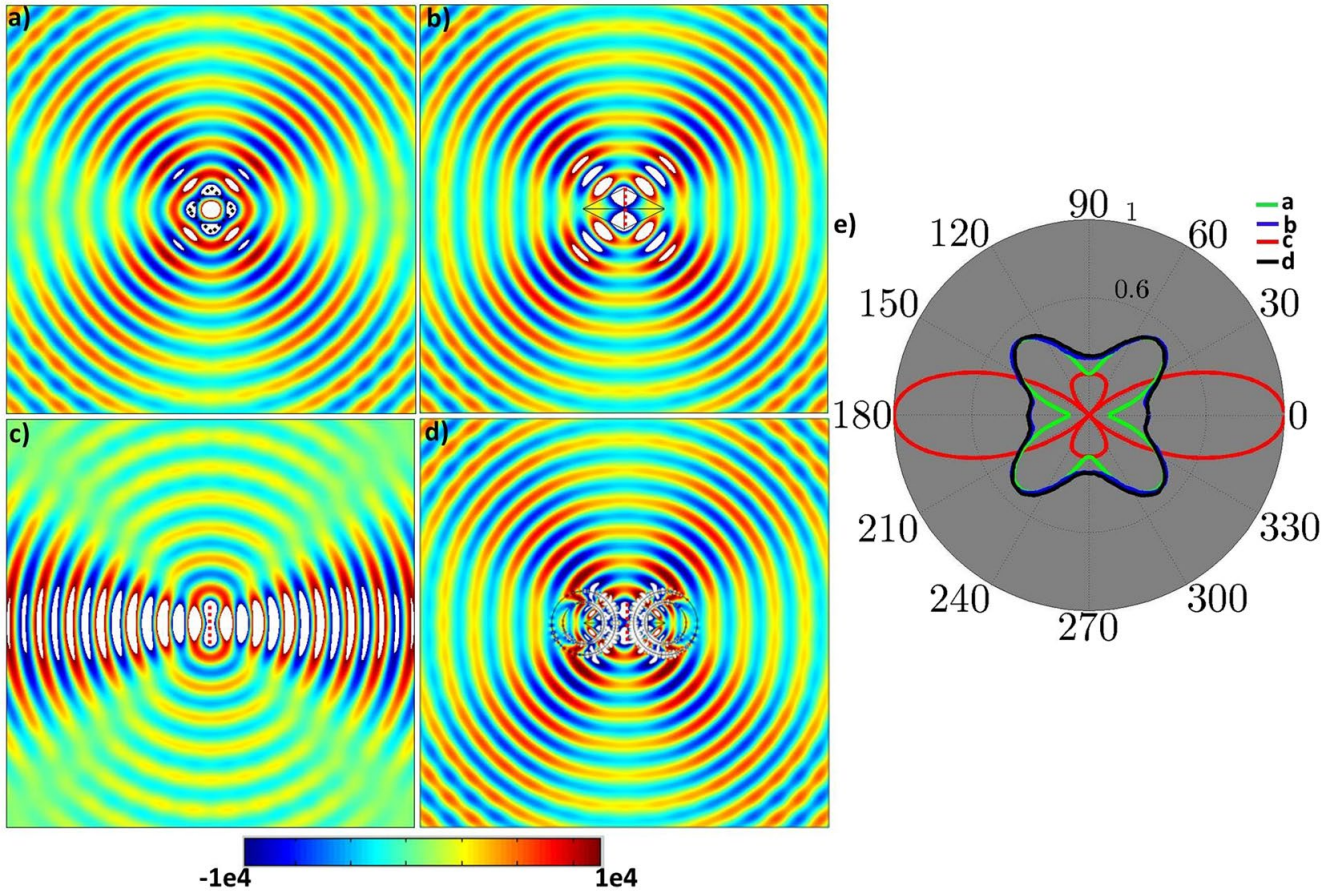
Simulation results of non-contact device for arrays of antenna. (**a**) A diamond shaped array of antennas is placed in free space. (**b**) The (**a**) is transformed into a linear array. (**c**) The linear array as of (**b**) is placed in free space without EM medium. (**d**) The proposed non-contact device is used to distantly change the radiation patterns of (**c**) to make it similar as that of (**b**) for the observers. (**e**) The normalized far-field radiation pattern of (**a**), (**b**), (**c**), and (**d**).

The simulation results are shown in Fig. 6(a–e), in which Fig. 6(a) shows the radiation pattern of diamond shaped antenna array in the free space and after first transformation, Fig. 6(b) represents the field pattern of linear antenna array surrounded by the referenced EM medium. Figure 6(c) demonstrates the radiation pattern of linear antenna array in free space. Finally, the proposed non-contact device is used in Fig. 6(d) to distantly change the radiations pattern of Fig. 6(c) according to Fig. 6(b). Figure 6(e) shows the normalized far-field radiation patterns of Fig. 6(a,b,c and d).

It should be noted that the practical implementation of the above-mentioned proposed devices becomes more limited due to the obtained material parameters of the designed devices. The proposed devices are composed by highly anisotropic parameters and with negative values in some region, simultaneously. However, with the help of metamaterials, we can achieve the required values, at least for certain polarization. In this regard, the split-ring resonators (SRRs) can achieve negative permeability, and the metal rod can achieve negative permittivity. By combining the both SRRs and metal rod^30,41^, the values can be achieved.

## Conclusion

In conclusion, we proposed three different devices to distantly and freely manipulate the radiation patterns of antennas with multi-folded TO method. The structure of proposed non-contact device contains a mapped EM medium embedded in the compressed region and covered by the folding region with negative index materials. We illustrate the proposed concept with detailed numerical simulations for each case of different proposed antenna devices and confirmed that the illusion of active scatterer can be distantly achieved. This proposed concept will be very helpful for future antenna design.

## Methods

The non-contact device (Fig. 1(d)) is designed through a homogeneous transformation method. For details, the transformation functions for compression of region III into region I are:

1 $$\begin{array}{c}x^{\prime} =\frac{a}{c}x+{x}_{0}(1-\frac{a}{c})\\ y^{\prime} =\frac{a}{c}y+{y}_{0}(1-\frac{a}{c})\\ z^{\prime} =z\end{array}$$

Then the constitutive parameters will become:

2 $${\varepsilon }_{I}={\mu }_{I}=diag[1,1,{(c/a)}^{2}]$$

Assuming the material parameters of referenced EM medium (blue colored) is $s=40y+3$, and then the parameters of its corresponding image in real space (green colored) will become:

3 $${\varepsilon }_{image}={\mu }_{image}=[1,1,{s}_{image}{(c/a)}^{2}]$$

where $${s}_{image}=40{y}_{image}+3$$, and $${y}_{image}=c/a[y^{\prime} -{y}_{0}+{y}_{0}a/c]$$.

Similarly, the transformation functions for the folding region II is:

4 $$\begin{array}{c}r^{\prime} =\frac{a-b}{c-b}r+\frac{c-a}{c-b}b\\ \theta ^{\prime} =\theta \\ z^{\prime} =z\end{array}$$

The constitutive parameters will become:

5 $${\varepsilon }_{II}={\mu }_{II}=diag[k{}_{1}{r}_{1}/r,k{}_{2}r/{r}_{1},k{}_{2}{r}_{1}/r]$$

where $${k}_{1}=(a-b)/(c-b)$$, $${k}_{2}=1/{k}_{1}$$, $$r=\sqrt{{(x-{x}_{0})}^{2}+{(y-{y}_{0})}^{2}}$$, $${r}_{1}=\frac{r(c-b)-b(c-a)}{a-b}$$, and $$\phi ={\tan }^{-1}((y-{y}_{0})/(x-{x}_{0}))$$.

For non-contact planar device (Fig. 3(d)), the transformation mapping contains two-steps. At first step, the parabolic antenna is transformed into a planar antenna with the material parameters obtained from ref.^25^. For ease, the transformation functions for the first step are given as:

6 $$\begin{array}{c}x^{\prime} =\frac{2pdx}{2p{a}_{1}-{y}^{2}}\\ y^{\prime} =y\\ z^{\prime} =z\end{array}$$

Then the constitutive parameters will become as:

7 $${\varepsilon }_{planar}={\mu }_{planar}=[\begin{array}{ccc}\frac{2({p}^{2}{d}^{2}+{x}^{2}{y}^{2})}{pd(2p{a}_{1}-{y}^{2})} & \frac{xy}{pd} & 0\\ \frac{xy}{pd} & \frac{2p{a}_{1}-{y}^{2}}{2pd} & 0\\ 0 & 0 & \frac{2p{a}_{1}-{y}^{2}}{2pd}\end{array}]$$

In the second step, the transformation functions and constitutive parameters are obtained from eqs (1–2) and eqs (4–5), for region I and region II respectively. Moreover, the material parameters of the image part in region I will become as

8 $${\varepsilon }_{pimage}={\mu }_{pimage}=diag[1,1,{\varepsilon }_{planar1}{(c/a)}^{2}]$$

Where ${\varepsilon }_{planar1}$ is obtained by replacing the $x$ and $y$ in eq. (7) with ${x}_{planar}$ and ${y}_{planar}$ respectively. where ${x}_{planar}=c/a[x^{\prime} -{x}_{0}+{x}_{0}a/c]$, and ${y}_{planar}=c/a[y^{\prime} -{y}_{0}+{y}_{0}a/c]$.

For third example, we consider only the first quadrant segment (blue colored) due to the symmetric structure of proposed device, which can be seen in Fig. 5(d). In the first step, the ${\rm{\Delta }}ABC$ is expanded to ${\rm{\Delta }}ABO$ with the following transformation equations:

9 $$\begin{array}{c}x^{\prime} =2x+2y-0.1\\ y^{\prime} =y\\ z^{\prime} =z\end{array}$$

In this way, the diamond shaped antenna arrays are transformed into a linear array with the material parameters of the first quadrant is: $${\varepsilon }_{{\rm{\Delta }}ABO}={\mu }_{{\rm{\Delta }}ABO}=[\begin{array}{ccc}4 & 1 & 0\\ 1 & 0.5 & 0\\ 0 & 0 & 0.5\end{array}]$$. In the second step, after recalling the Fig. 1(d), the material parameters will turn to $${\varepsilon }_{{\rm{\Delta }}ABO(image)}={\mu }_{{\rm{\Delta }}ABO(image)}=[\begin{array}{ccc}4 & 1 & 0\\ 1 & 0.5 & 0\\ 0 & 0 & 0.5{(c/a)}^{2}\end{array}]$$, for the same segment. Hereafter, eqs (4–5) are used to obtain the constitutive parameters for the folding region.

## References

[1] Leonhardt U (2006). Optical conformal mapping. Science.

[2] Pendry JB, Schurig D, Smith DR (2006). Controlling electromagnetic fields. Science.

[3] Rahm M (2008). Design of electromagnetic cloaks and concentrators using form-invariant coordinate transformations of Maxwell’s equations. Photon. Nanostruct. Fundam. Appl..

[4] Rahm M (2008). Transformation-optical design of adaptive beam bends and beam expanders. Opt. Express.

[5] Rahm M (2008). Optical design of reflectionless complex media by finite embedded coordinate transformations. Phys. Rev. Lett..

[6] Lin L (2008). Electromagnetic concentrators with reduced material parameters based on coordinate transformation. Opt. Express.

[7] Huangfu J (2008). Application of coordinate transformation in bent waveguides. J. Appl. Phys..

[8] Tichit P-H (2010). Waveguide taper engineering using coordinate transformation technology. Opt. Express.

[9] Wang H (2017). Panoramic lens designed with transformation optics. Sci. Rep..

[10] Ma HF (2010). Three-dimensional broadband and broad-angle transformation-optics lens. Nat. Commun..

[11] Yi J (2015). Restoring in-phase emissions from non-planar radiating elements using a transformation optics based lens. Appl. Phys. Lett..

[12] Tichit P-H (2009). Ultradirective antenna via transformation optics. J. Appl. Phys..

[13] Luo Y (2009). High-directivity antenna with small antenna aperture. Appl. Phys. Lett..

[14] Tichit P-H (2011). Design and experimental demonstration of a high-directive emission with transformation optics. Phys. Rev. B.

[15] Jiang ZH (2011). Experimental demonstration of a broadband transformation optics lens for highly directive multibeam emission. Phys. Rev. B.

[16] Jiang ZH (2012). Broadband high directivity multibeam emission through transformation optics-enabled metamaterial lenses. IEEE Trans. Antennas Propag..

[17] Tichit P-H (2014). Spiral-like multi-beam emission via transformation electromagnetics. J. Appl. Phys..

[18] Tichit P-H (2011). Transformation media producing quasi-perfect isotropic emission. Opt. Express.

[19] Tichit P-H (2013). Experimental verification of isotropic radiation from a coherent dipole source via electric-field-driven LC resonator metamaterials. Phys. Rev. Lett..

[20] Qian C (2016). Transient response of a signal through a dispersive invisibility cloak. Opt. Lett..

[21] Yang Y (2016). Full‐Polarization 3D Metasurface Cloak with Preserved Amplitude and Phase. Adv. Mater..

[22] Li R (2017). Design of Ultracompact Graphene-Based Superscatterers. IEEE. J. Sel. Top. Quant..

[23] Kwon, D.-H. Transformation Electromagnetic Design of an Embedded Monopole in a Ground Recess for Conformal Applications. *IEEE Antenn. Wirel. Pr*. **9** (2010).

[24] Popa, B.-l. *et al*. Conformal array design with transformation electromagnetics. *Appl. Phys. Lett*. **94**, 244102 (2009).

[25] Kong F (2007). Planar focusing antenna design by using coordinate transformation technology. Appl. Phys. Lett..

[26] Lu W (2009). Transformation media based super focusing antenna. J. Phys. D: Appl. Phys..

[27] Zhang J (2008). Manipulating the directivity of antennas with metamaterial. Opt. Express.

[28] Luo, Y. *et al*. New Concept Conformal Antennas Utilizing Metamaterial and Transformation Optics. *IEEE Antenn. Wirel. Pr*. **7** (2008).

[29] Lai Y (2009). Illusion optics: The optical transformation of an object into another object. Phys. Rev. Lett..

[30] Zheng B (2016). Concealing arbitrary objects remotely with multi-folded transformation optics. Light-Sci. Appl..

[31] Jiang WX (2010). Illusion media: Generating virtual objects using realizable metamaterials. Appl. Phys. Lett..

[32] Li C (2010). Experimental realization of a circuit-based broadband illusion-optics analogue. Phys. Rev. Lett..

[33] Jiang WX (2011). Radar illusion via metamaterials. Phys. Rev. E.

[34] Jiang WX (2011). Shrinking an arbitrary object as one desires using metamaterials. Appl. Phys. Lett..

[35] Jiang W (2013). Creation of ghost illusions using wave dynamics in metamaterials. Adv. Funct. Mater..

[36] Tichit P-H (2014). Transformation electromagnetics for antennas with an illusion on the radiation pattern. IEEE Antennas Wireless Propag. Lett..

[37] Yi J (2015). Illusion optics: Optically transforming the nature and the location of electromagnetic emissions. J. Appl. Phys..

[38] Madni HA (2016). Non-contact radio frequency shielding and wave guiding by multi-folded transformation optics method. Sci. Rep..

[39] Han T (2010). An arbitrarily shaped cloak with nonsingular and homogeneous parameters designed using a twofold transformation. J. Opt..

[40] Han T (2010). Distributed external cloak without embedded antiobjects. Opt. Lett..

[41] Shelby RA (2001). Experimental verification of negative index of refraction. Science.

